# A Rare Case of Esophageal Adenocarcinoma with Urinary Bladder Metastasis

**DOI:** 10.1155/2017/9343896

**Published:** 2017-05-31

**Authors:** Heather Katz, Rahoma E. Saad, Krista Denning, Toni O. Pacioles

**Affiliations:** ^1^Department of Hematology/Oncology, Joan C. Edwards School of Medicine, Marshall University, 1600 Medical Center Dr, Huntington, WV 25701, USA; ^2^Department of Internal Medicine, Joan C. Edwards School of Medicine, Marshall University, 1600 Medical Center Dr, Huntington, WV 25701, USA; ^3^Department of Pathology, Joan C. Edwards School of Medicine, Marshall University, 1600 Medical Center Dr, Huntington, WV 25701, USA

## Abstract

Metastatic esophageal adenocarcinoma to the urinary bladder is extremely rare. We describe a previously healthy 49-year-old female with recent diagnosis of adenocarcinoma of the gastroesophageal junction with metastatic disease to the liver. Biopsy was positive for human epidermal growth factor receptor 2 (HER2) by Fluorescence In Situ Hybridization (FISH). She received six cycles of Cisplatin, 5-Fluorouracil, and Herceptin and subsequently developed symptomatic anemia and hematuria. Cystoscopy with retroflexion was performed and she received a transurethral resection of bladder tumor with fulguration. Pathology of the bladder tumor revealed similar morphology to her liver metastasis and immunohistochemical stains were consistent with metastatic esophageal cancer. Three weeks after being diagnosed with metachronous urinary bladder metastasis from esophageal adenocarcinoma primary, she expired. She only received her first cycle of palliative chemotherapy with Ramucirumab and Paclitaxel.

## 1. Introduction

Esophageal cancer is the eighth most common cancers and the sixth cause of all cancer death worldwide [1]. The two main histological subtypes of esophageal cancer are squamous cell carcinoma (SCC) and adenocarcinoma [1–4]. Esophageal cancer is a highly fatal malignancy [1–4]. In United States, the overall 5-year survival rate for esophageal adenocarcinoma is less than 20% [1–5]. The high mortality from esophageal cancer is attributed to local invasion and distant spread of this malignancy [1–5]. Metastasis of esophageal cancer usually occurs to regional lymph nodes, lungs, liver, brain, bone, and peritoneum [6, 7]. Distant metastasis of esophageal cancer to urinary bladder is extremely rare and has only been reported twice worldwide [7, 8].

We report the third case of metastasis to the urinary bladder from the esophagus; however this is the first case of metachronous metastasis despite being treated previously with six cycles of chemotherapy with Cisplatin, 5-Fluorouracil, and Trastuzumab.

## 2. Case Report

A 49-year-old female with a past medical history of gastroesophageal reflux disease, anemia, and anxiety presented initially with symptoms of early satiety, feeling bloated, fullness, vomiting, dysphagia to solids, and a 36-pound weight loss over the past 6 months. She has a 35-pack year history of smoking. There is no family history of cancer. On physical exam, her vitals were stable and her exam was pertinent for cachexia and paleness of the skin and conjunctiva. Labs revealed iron deficiency anemia with hemoglobin of 10 g/dL and elevated alkaline phosphatase 171 units/L.

**Figure 1:**
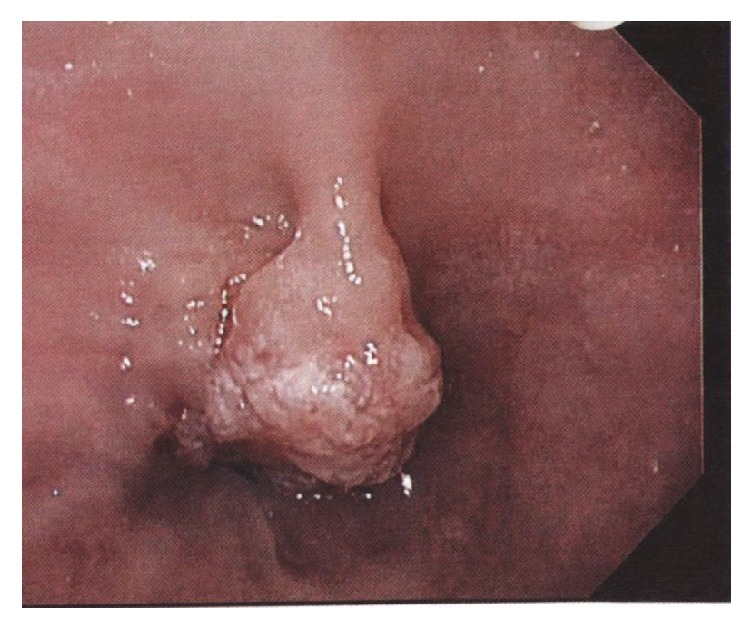
Esophageal tumor seen during EGD.

**Figure 2:**
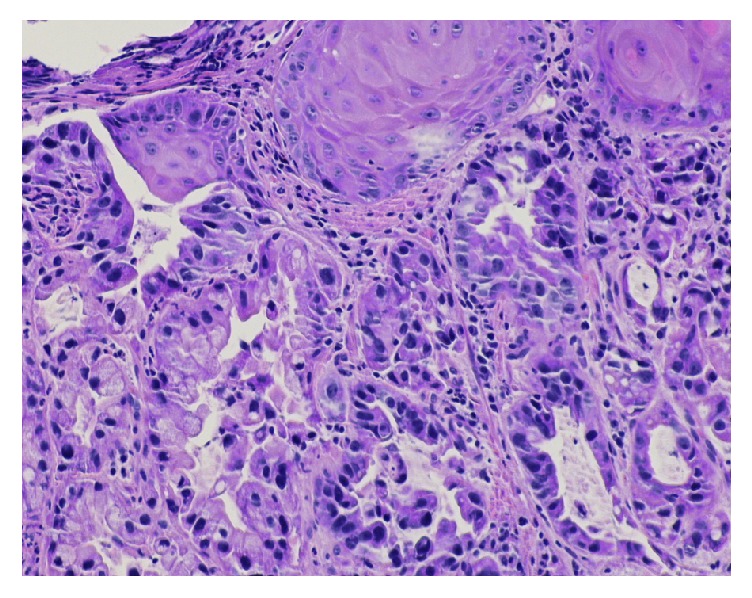
H&E stain of the esophageal tumor showing adenocarcinoma.

**Figure 3:**
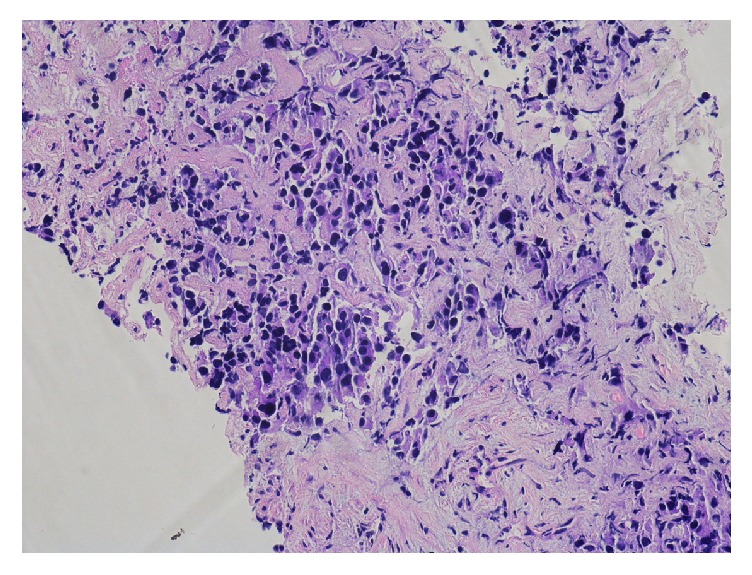
H&E stain from liver biopsy with morphologically similar cells to the esophagus biopsy.

Due to her complaints and findings of iron deficiency, esophagogastroduodenoscopy (EGD) was done and showed a mass at the distal esophagus causing a tight stricture (Figure 1). The biopsy showed adenocarcinoma of the esophagus (Figure 2). Endoscopic ultrasound revealed a hypoechoic mass that was seen at the junction invading all the layers of the esophageal wall up to the capsule of the liver. Two 6.5 mm lymph nodes were seen around the mass due to the stricture. Further staging was performed with computed tomography (CT) chest, abdomen, and pelvis. CT scan of the chest with contrast showed mild thickening of the distal esophagus without other findings. CT scan of the abdomen and pelvis revealed retroperitoneal adenopathy most prominent in the para-aortic location measuring up to 1.9 × 1.7 cm and extending along the internal and external iliac chain as well as hypodense lesions within the liver. The liver lesions were biopsied and the cells were morphologically similar to the cells from the esophageal tumor (Figure 3). Final pathology revealed poorly differentiated adenocarcinoma, consistent with patient known diagnosis of adenocarcinoma of the esophagus.

HER2 immunohistochemical staining was negative with weak (1+) discontinuous membranous staining; however Fluorescence In Situ Hybridization (FISH) for HER2 was performed and was positive, with HER2 amplified HER2/CN17 ratio: 3.8.

Positron emission tomography scan was performed 6 months after diagnosis showed a hypermetabolic subcarinal lymph node showing a peak standardized uptake value (SUV) of 5 and diffuse hypermetabolic activity involving the distal esophagus into the proximal stomach, peak SUV 13.0. There was also a hypermetabolic mass in the right lobe of the liver measuring approximately 3 cm in size showing a peak SUV of 11.0. There were no other areas of abnormal radiotracer accumulation.

She completed six cycles of Cisplatin, 5-Fluorouracil, and Herceptin; however prior to the last cycle the patient was found to have symptomatic anemia and upon further questioning hematuria.

**Figure 4:**
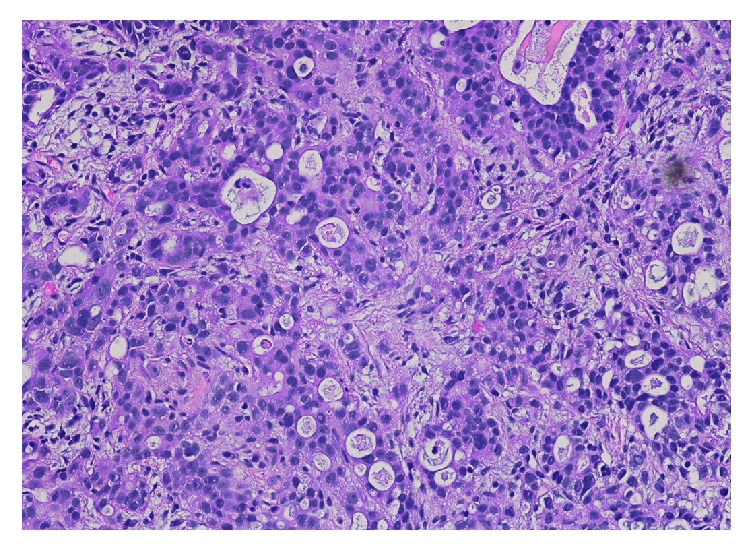
H&E stain of the bladder tumor with morphologically similar cells to the esophagus biopsy.

**Figure 5:**
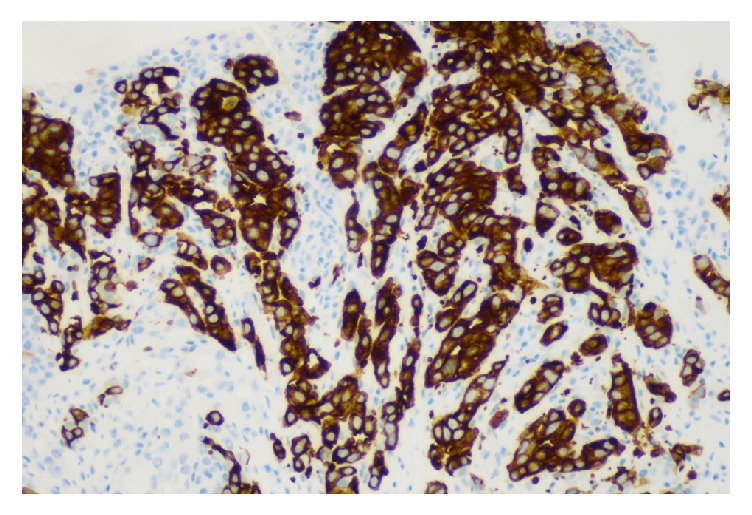
Bladder tumor with CK20 positive stain.

**Figure 6:**
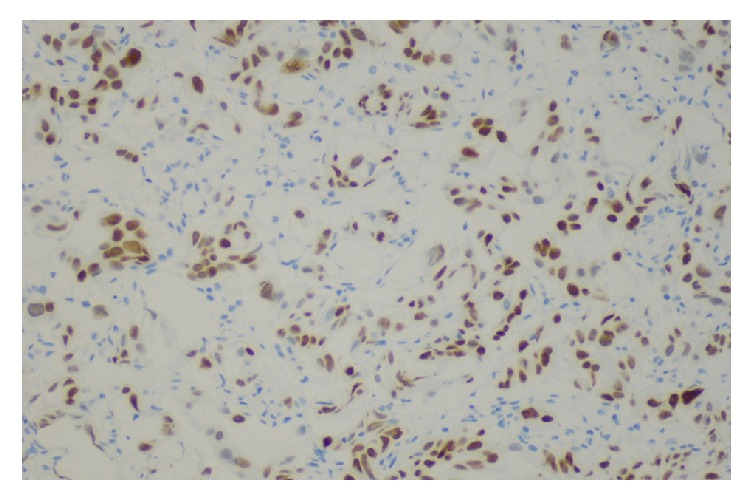
Bladder tumor with positive stain for CDX2.

**Figure 7:**
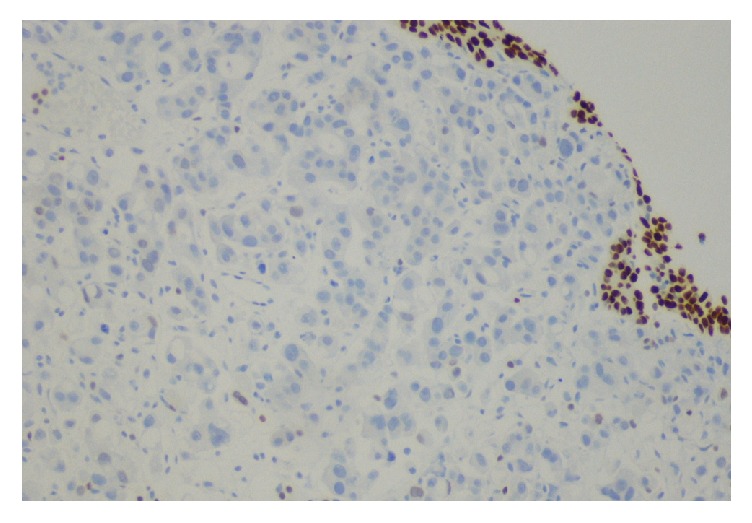
Bladder tumor with negative stain for GATA 3 in neoplastic cells.

**Figure 8:**
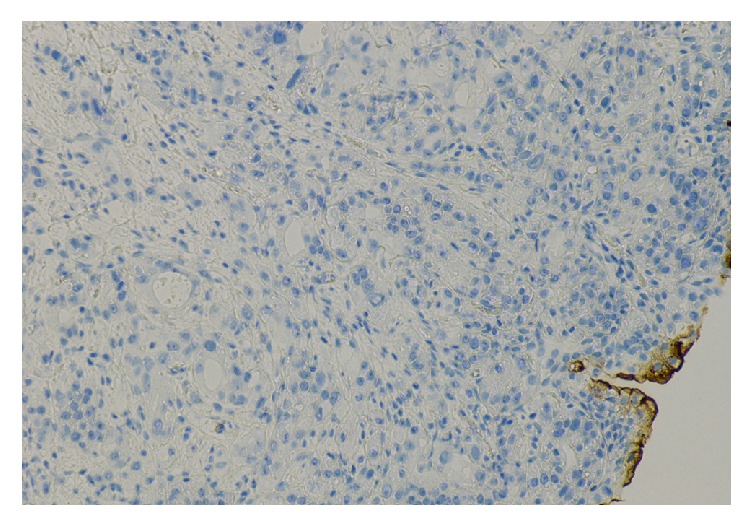
Bladder tumor with negative stain for Uroplakin in neoplastic cells.

A cystoscopy with retroflexion and transurethral resection of bladder tumor with fulguration was performed. Pathology was compared to the previous biopsy of the liver and was thought to be morphologically similar (Figure 4). Immunohistochemical (IHC) stains showed cytokeratin 7 positive, cytokeratin 20 positive (Figure 5), Caudal Type Homeobox 2 (CDX2) positive (Figure 6), GATA3 negative in neoplastic cells (Figure 7), and Uroplakin negative in neoplastic cells (Figure 8). The morphologic and immunohistochemical findings were consistent with metastatic esophageal carcinoma. The patient was then started on palliative immunotherapy and chemotherapy with Ramucirumab and Paclitaxel and completed one cycle before receiving hospice care and ultimately expiring one week later.

## 3. Discussion

Esophageal cancer is the eighth most common cancer and the sixth cause of all cancer deaths worldwide [1]. While squamous cell carcinoma of the esophagus is the most common histological subtype of esophageal cancer globally, in western countries, adenocarcinoma has become the predominant subtype [1–5]. Risk factors for adenocarcinoma of the esophagus include gastroesophageal reflux disease (GERD), obesity, and smoking [1–5].

There are 5 main routes of metastasis of esophageal and gastric cancer: (1) direct invasion; (2) lymphatic spread; and (3) hematogenous, (4) transperitoneal, and (5) intraluminal implantation [6]. The most common areas of esophageal cancer metastasis are to the liver and peritoneum [6–8], regional lymph nodes, lung, pleura, stomach, kidney, adrenals, and bone [8]. The urinary bladder is an extremely rare site of metastasis. Velcheti and Govindan [7] reviewed 264 cases of metastatic disease to the bladder and found the most common primary site to be genitourinary, colorectal, melanoma, breast, stomach, unknown primary, choriocarcinoma, lung, and pancreas. After an extensive literature search, there have only been two cases of metastatic disease to the bladder originating from the esophagus described in the literature.

Schuurman et al. [8] described a case of a 53-year-old male who presented with abdominal pain and difficulty in swallowing. He received an EGD with biopsy and was diagnosed with adenocarcinoma of the esophagus. Metastatic workup showed no obvious distant metastasis; however a thickened dorsal bladder wall was noted and biopsy revealed malignant tumor consistent with adenocarcinoma of the esophagus. Matsumoto et al. [9] described a case of a 74-year-old man presenting with gross hematuria and workup revealed an intrapelvic tumor invading the bladder, rectum, sigmoid colon, and left ilium. Pathologic diagnosis of the intrapelvic tumor was moderately differentiated squamous cell carcinoma. Preoperatively, gastrointestinal fiberscopy with biopsy revealed moderately differentiated squamous cell carcinoma. The intrapelvic tumor that invaded the bladder was diagnosed as metastatic tumor from esophageal cancer. While the cases described by Schuurman et al. and Matsumoto et al. discussed synchronous bladder metastasis at time of diagnosis, our patient had metachronous bladder metastasis despite completing a course of chemotherapy for esophageal cancer with liver metastasis.

Immunohistochemical staining is essential to capture the correct diagnosis and establish metastatic versus primary disease. In the present case, immunohistochemistry (IHC) stained positive for CK7, CK20, and CDX2 and negative for Uroplakin and GATA3 (Table 1). This confirmed the diagnosis of esophageal adenocarcinoma with metastasis to urinary bladder and excluded primary adenocarcinoma in the bladder which was crucial to ensure appropriate treatment. Gastrointestinal tumors stain for cytokeratins 7 and 20, or CDX-2, a homeobox gene protein expressed in nuclei of the intestinal epithelium that functions as a tumor suppressor gene [10]. CDX-2 has been shown to be sensitive and specific for gastrointestinal tumors, including esophageal adenocarcinoma [11]. This marker is absent in bladder carcinoma. High-molecular weight cytokeratins, cytokeratin 7, cytokeratin 20, and GATA-3 have previously been used to diagnose bladder cancer [12]. A specific antigen such as Uroplakin III appears specific to urothelial origin and has been used more recently [13].

This is the first case of metachronous metastatic esophageal cancer to the urinary bladder and will be a useful adjunct to the current literature. This case highlights the importance of comprehensive evaluations and further workups in patients being treated for metastatic esophageal cancer who present with hematuria and symptomatic anemia. Although rare, in patients with esophageal cancer who present with these symptoms, metastatic disease from an esophageal primary tumor should be in the differential diagnosis.

## Figures and Tables

**Table 1:** Immunohistochemistry stains used to diagnose primary esophageal cancer, metastasis from an esophageal primary cancer, and primary bladder cancer.

Immunohistochemistry stains	Esophageal tumor	Liver biopsy (neoplastic cells)	Bladder biopsy (neoplastic cells)	*Primary *bladder cancer
CK7	+	+	+	+
CK20	+	+	+	+
CDX-2	+	+	+	−
GATA-3	−	−	−	+
Uroplakin	−	−	−	+

## References

[1] Napier K. J., Scheerer M., Misra S. (2014). Esophageal cancer: a review of epidemiology, pathogenesis, staging workup and treatment modalities. *World Journal of Gastrointestinal Oncology*.

[2] Pohl H., Sirovich B., Welch H. G. (2010). Esophageal adenocarcinoma incidence: are we reaching the peak?. *Cancer Epidemiology Biomarkers and Prevention*.

[3] Buas M. F., Vaughan T. L. (2013). Epidemiology and risk factors for gastroesophageal junction tumors: understanding the rising incidence of this disease. *Seminars in Radiation Oncology*.

[4] Engel L. S., Chow W., Vaughan T. L. (2003). Population attributable risks of esophageal and gastric cancers. *JNCI Journal of the National Cancer Institute*.

[5] Dubecz A., Gall I., Solymosi N. (2012). Temporal trends in long-term survival and cure rates in esophageal cancer: a SEER database analysis. *Journal of Thoracic Oncology*.

[6] Makker J., Karki N., Sapkota B., Niazi M., Remy P. (2016). Rare presentation of gastroesophageal carcinoma with rectal metastasis: a case report. *American Journal of Case Reports*.

[7] Velcheti V., Govindan R. (2007). Metastatic cancer involving bladder: a review. *The Canadian Journal of Urology*.

[8] Schuurman J. P., De Vries Reilingh T. S., Roothaan S. M., Bijleveld R. T., Wiezer M. J. (2009). Urinary bladder metastasis from an esophageal adenocarcinoma: a case report. *American Journal of Gastroenterology*.

[9] Matsumoto Y., Mibu H., Kagebayashi Y., Miyasaka Y. (2004). Metastatic intrapelvic tumor from esophageal cancer: a case report. *Hinyokika Kiyo*.

[10] Suh E., Chen L., Taylor J., Traber P. G. (1994). A homeodomain protein related to caudal regulates intestine-specific gene transcription. *Molecular and Cellular Biology*.

[11] Phillips R. W., Frierson H. F., Moskaluk C. A. (2003). Cdx2 as a marker of epithelial intestinal differentiation in the esophagus. *American Journal of Surgical Pathology*.

[12] Amin M. B., Trpkov K., Lopez-Beltran A., Grignon D., Members of the ISUP Immunohistochemistry in Diagnostic Urologic Pathology Group (2014). Best practices recommendations in the application of immunohistochemistry in the bladder lesions: report from the International Society of Urologic Pathology consensus conference. *The American Journal of Surgical Pathology*.

[13] Kaufmann O., Volmerig J., Dietel M. (2000). Uroplakin III is a highly specific and moderately sensitive immunohistochemical marker for primary and metastatic urothelial carcinomas. *American Journal of Clinical Pathology*.

